# Supplementary material for: Virtual reality as a tool for environmental conservation and fundraising
Source: PLoS One. 2020 Apr 6;15(4):e0223631. doi: 10.1371/journal.pone.0223631 (PMC7135095; doi:10.1371/journal.pone.0223631)
Supplement: S1 File — (PDF) [file pone.0223631.s001.pdf]

GILI ECO TRUST

ZMT LEIBNIZ CENTRE for Tropical Marine Research

Respondent ID:
Intern name:

Agreement to Participate

Researcher: Katherine Nelson   Contact: katie.nelson@leibniz-zmt.de

This is a research project on communication for coral reef conservation. You will observe a short video and then complete a survey questionnaire. This research has been approved by the Leibniz ZMT ethical review committee in accordance with the German Data Protection Act. **Your responses are anonymous. No information that could identify you will be included in any reports of this study.**

By signing this form, you are giving voluntary consent to participate in this study. You may refuse to participate in the entire study or in any part of the study. If you decide to participate in the study, you are free to withdraw at any time.

_________________________________ ____________________

Participant's Signature   Date

Do you agree that we can use your picture in the future when presenting the results of the study? YES ☐ NO ☐

Questionnaire

You have a ten percent chance of earning 100.000 Rupiah for completing the survey. If you are chosen as the person that receives this money, how much (if any) would you like to donate to Yayasan Ekosistem Gili Indah marine conservation organization?

I would like to donate Rupiah…

☐0k ☐10k ☐20k ☐30k ☐40k ☐50k ☐60k ☐70k ☐80k ☐90k ☐100k

Please answer the following questions. Only your opinion is important – there are no correct or incorrect answers. Please don't think for long, but answer spontaneously. Check the box which applies most.

	Yes						No
Q2: In this moment, do you feel sad?	☐	☐	☐	☐	☐	☐	☐
Q3: In this moment, do you feel happy?	☐	☐	☐	☐	☐	☐	☐
Q4: In this moment, do you feel helpless?	☐	☐	☐	☐	☐	☐	☐
Q5: In this moment, do you feel hopeful?	☐	☐	☐	☐	☐	☐	☐
Q6: Did you feel like you were part of the underwater world?	☐	☐	☐	☐	☐	☐	☐
Q7: Did you feel like you were surrounded by corals and fish?	☐	☐	☐	☐	☐	☐	☐
Q8: Did it feel like you were really under water?	☐	☐	☐	☐	☐	☐	☐
Q9: Does hearing about environmental degradation make you reflective?	☐	☐	☐	☐	☐	☐	☐
Q10: Does global climate change worry you?	☐	☐	☐	☐	☐	☐	☐
Q11: Do you think that global climate change will have severe consequences?	☐	☐	☐	☐	☐	☐	☐
Q12: Do you think that global climate change will have severe consequences for you personally?	☐	☐	☐	☐	☐	☐	☐

	Yes						No
Q13: I prefer buying new things rather than fixing my broken things	☐	☐	☐	☐	☐	☐	☐
Q14: I prefer to eat meals with meat/fish rather than vegetable only	☐	☐	☐	☐	☐	☐	☐
Q15: I bring my own reusable bag to the market	☐	☐	☐	☐	☐	☐	☐
Q16: I recycle (e.g., papers, cans, bottles, batteries)	☐	☐	☐	☐	☐	☐	☐

	Yes						No
Q17: I bring my own container for take out food	☐	☐	☐	☐	☐	☐	☐
Q18: I volunteer time to help an environmentalist group	☐	☐	☐	☐	☐	☐	☐
Q19: I use single use plastic (e.g. straws, plastic bags, water bottles)	☐	☐	☐	☐	☐	☐	☐
Q20: I make travel plans based on the amount of CO2 emissions generated by the vehicles I use	☐	☐	☐	☐	☐	☐	☐
Q21: I purchase recycled materials even if they are more expensive	☐	☐	☐	☐	☐	☐	☐
Q22: I only buy products from companies that have a strong record of protecting the environment	☐	☐	☐	☐	☐	☐	☐
	Optimistic						Pessimistic
Q23: Optimists are people who look ahead with confidence and mostly expect good results. Pessimists are people who look ahead doubtfully and mostly expect bad results. Please assess yourself: In respect to global climate change, where do you fall on the spectrum from optimism to pessimism?	☐	☐	☐	☐	☐	☐	☐
	Yes						No
Q24: Did you ever donate money to an environmental protection organization?	☐						☐
Q25: Did you ever see real coral reefs in the ocean?	☐						☐
Q26: Are you a member of an environmental organization?	☐						☐
	Lowest						Highest
Q27: Please mark where you think you and your family are on the income spectrum in the country where you're from.	☐	☐	☐	☐	☐	☐	☐
Q28: Where do you think you are in terms of environmental consciousness compared to other people your age?	☐	☐	☐	☐	☐	☐	☐
Q29: Assess your level of generosity in comparison to other people your age.	☐	☐	☐	☐	☐	☐	☐
	No focus on sustainability						Completely sustainable
Q30: In the future, where do you see yourself on the spectrum of living a sustainable and environmentally conscious lifestyle.	☐	☐	☐	☐	☐	☐	☐
	Not religious						Most religious
Q31: Please assess where you are on the spectrum of religiosity.	☐	☐	☐	☐	☐	☐	☐

Q32: Nationality:.. **Q33:How far is the place where you grew up from the coast (in km)**..........................

Q34: Age: ------------- **Q35: Gender** ☐ **Female** ☐ **Male** ☐ **Other** **Q36: Field of study/profession:** -------------------------

Q37: Please circle your highest level of education: 1) Elementary school (first 6 years) 2) Secondary school (7-9 years of school) 3) Senior high school (10-12 years) 4) Vocational school (Trade apprentice) 5) Bachelors degree 6) Post-graduate degree
